# Genome-wide SNP discovery and QTL mapping for fruit quality traits in inbred backcross lines (IBLs) of *solanum pimpinellifolium* using genotyping by sequencing

**DOI:** 10.1186/s12864-016-3406-7

**Published:** 2017-01-03

**Authors:** Ibrahim Celik, Nergiz Gurbuz, Ali Tevfik Uncu, Anne Frary, Sami Doganlar

**Affiliations:** 1 Department of Molecular Biology and Genetics, Izmir Institute of Technology, Urla, Izmir Turkey; 2 Pressent Address: Department of Molecular Biology and Genetics, Necmettin Erbakan University, Konya, Turkey

**Keywords:** Fruit weight, pH, Soluble solids content, Tomato genome

## Abstract

**Background:**

*Solanum pimpinellifolium* has high breeding potential for fruit quality traits and has been used as a donor in tomato breeding programs. Unlocking the genetic potential of *S. pimpinellifolium* requires high-throughput polymorphism identification protocols for QTL mapping and introgression of favourable alleles into cultivated tomato by both positive and background selection.

**Results:**

In this study we identified SNP loci using a genotyping by sequencing (GBS) approach in an IBL mapping population derived from the cross between a high yielding fresh market tomato and *S. pimpinellifolium* (LA1589) as the recurrent and donor parents, respectively. A total of 120,983,088 reads were generated by the Illumina HiSeq next-generation sequencing platform. From these reads 448,539 sequence tags were generated. A majority of the sequence tags (84.4%) were uniquely aligned to the tomato genome. A total of 3.125 unique SNP loci were identified as a result of tag alignment to the genome assembly and were used in QTL analysis of 11 fruit quality traits. As a result, 37 QTLs were identified. *S. pimpinellifolium* contributed favourable alleles for 16 QTLs (43.2%), thus confirming the high breeding potential of this wild species*.*

**Conclusions:**

The present work introduced a set of SNPs at sufficiently high density for QTL mapping in populations derived from *S. pimpinellifolium* (LA1589). Moreover, this study demonstrated the high efficiency of the GBS approach for SNP identification, genotyping and QTL mapping in an interspecific tomato population.

**Electronic supplementary material:**

The online version of this article (doi:10.1186/s12864-016-3406-7) contains supplementary material, which is available to authorized users.

## Background

*Solanum pimpinellifolium* has high breeding potential for biotic and abiotic stress tolerance and fruit quality traits such as fruit weight, internal and external color, firmness, fruit shape, lycopene and high soluble solids content. As a result, this wild species has been frequently used as a donor in tomato breeding programs [1–6]. In addition, the *S. pimpinellifolium* genome is reported to harbor more favorable and fewer unfavorable alleles for breeding improved cultivars compared to other wild tomato species including *S. cheesmanii, S. chmielewskii* and *S. habrochaites* [7]. *S. pimpinellifolium* is more closely related to cultivated tomato (*Solanum lycopersicum)* than the other wild tomato species*.* Thus, utilization of *S. pimpinellifolium* in breeding minimizes the consequences of linkage drag [1]. Efficient introgression of favorable alleles from *S. pimpinellifolium* into *S. lycopersicum* by both positive and background selection requires high-throughput marker identification and genotyping as well as identification of the QTLs (Quantitative Trait Loci) that control the traits of interest [8].

Cultivated tomato has extensive genomic resources for molecular breeding. The genome has been completely sequenced and a large amount of data about genome-wide intraspecific and interspecific allelic variation are available. More than 20 interspecific tomato linkage maps were constructed using SSR (Simple Sequence Repeats), COSII (Conserved Ortholog Set II) and SNP (Single Nucleotide Polymorphism) markers. Nine of these maps were constructed using mapping populations derived from crosses between *S. pimpinellifolium* and *S. lycopersicum* for mapping of fruit quality traits and disease tolerance (reviewed by Foolad [9]). Most of these low resolution linkage maps were constructed with RFLP (Restriction Fragment Length Polymorphism), COSII and SSR markers. Four recently constructed linkage maps contain SNP markers designed from the Solanaceae Genomics Network [10] and Tomato Mapping Resource databases [11–14]. The maps introduced by Salinan et al. [13] and Capel et al. [14], respectively, contained 112 and 233 SNP markers genotyped in *S. pimpinellifolium* and *S. lycopersicum* using the high-resolution melting (HRM) method. Another map with 440 markers was constructed with SNPs discovered by Restriction site Associated DNA Sequencing (RAD-Seq) technology and VeraCode SNP makers [15]. The TraitGenetics EXPIMP2012 linkage map had the highest number of interspecific SNP markers (4,491 SNPs) [12]. Two additional studies have described development of SNP markers by sequencing. In the work of Causse et al. [16], eight (four cherry-type and four cultivars) *S. lycopersicum* accessions were sequenced to identify 16,000 unique, non-synonymous SNPs and 1,686 putative copy-number variations (CNVs). The other study used sequencing data available from the NCBI database for nine tomato accessions (two resequenced genomes and seven transcriptomes and yielded 4,680,647 SNPs [17]. However, none of the SNPs from these two projects were validated in segregated populations. Genotyping by sequencing (GBS) [18] is a practical and inexpensive method for high-throughput SNP discovery and genotyping. The GBS approach uses next-generation sequencing (NGS) technologies for multiplex sequencing of restriction site-associated DNA [16]. Alignment of the sequence reads generated from a population enables simultaneous SNP discovery and genotyping [18–20]. To date, the GBS approach has been commonly used for SNP discovery and genotyping in crop species such as wheat [21], rice [22] and maize [23]. However, the RAD-Seq method, which is similar to GBS has been used in tomato [15]. This technique was used to discover 4697 SNPs in an F2:3 population derived from a cross between *S. pimpinellifolium* (L3708) and *S. lycopersicum* (T3224) to identify QTLs for late blight resistance [15, 18]). The primary goal of the current study was to use the GBS approach for high-throughput identification of interspecific SNPs in the *S. pimpinellifolium* (LA1589) and *S. lycopersicum* cv. Tueza genomes. The second aim of this work was the identification of QTLs for fruit quality traits using the identified markers. To achieve these aims, an IBL (Inbred Backcross Line) population (BC_2_F_6_) comprising 93 individuals was developed using an advanced backcross (AB) QTL strategy. The donor parent was the wild tomato species *S. pimpinellifolium* (LA1589) and the recurrent parent was an elite fresh market tomato, *S. lycopersicum* cv. Tueza. IBL populations are used to introgress favorable alleles from wild species into the cultivated genome in tomato breeding. Although unbalanced populations such as IBLs are not suitable for linkage map construction due to unequal representation of the parental genomes, they are valuable genetic resources for QTL mapping because IBL populations contain small introgressions from the wild species in the cultivated tomato genome. Such small introgressions and the higher occurrence of crossing-over events as compared to F_2_ and BC populations improve the resolution of QTL mapping in IBL populations [24].

Fruit quality traits such as fruit weight, external and internal color, and firmness affect consumer preference and define the market value of tomato cultivars. Thus, tomato breeders are interested in the improvement of tomato cultivars for these traits and several studies were performed to identify QTLs controlling them [1, 2, 14, 25–28]. The most comprehensive QTL mapping study for tomato fruit quality traits was performed by Doganlar et al. [3]. In this study, 71 QTLs were identified for 22 fruit quality traits using an IBL population derived from the cross between *S. pimpinellifolium* (LA1589) and a processing tomato cultivar (*S. lycopersicum* cv. M82). Despite the high number of loci identified for fruit quality traits, most QTL analyses were performed using low resolution genetic linkage maps and transient populations, with the notable exceptions of work with IBLs [3] and RILs [13].

In the present study, QTLs were identified for 11 fruit quality traits (fruit weight, dry matter weight, external color, internal color, locule number, wall thickness, firmness, fruit shape, stem scar, soluble solids content, and pH) in an IBL population using interspecific SNP loci discovered through a GBS approach. This is the first time that GBS methodology was implemented in tomato for generation of a high resolution physical map for QTL mapping of fruit quality traits. The results of the present study demonstrate the efficiency of GBS for SNP discovery and QTL mapping in tomato.

## Methods

### Plant materials

An interspecific IBL (inbred backcross line) population derived from the cross *S. lycopersicum* cv. Tueza x *S. pimpinellifolium* (LA1589) was used as plant material in the study. Tueza is a cultivated fresh market tomato line with large (150–160 g), red, slightly flattened round fruits. LA1589 is a wild type tomato with small, red, round fruits. The IBL population and parents were grown by Multi Tohum seed company (Antalya, Turkey). A total of 10 plants per genotype were grown in double rows with 140 cm between wide rows and 50 cm between narrow rows. Within rows, plants were spaced at 40 cm intervals. For basal fertilization, 500 kg 15:15:15 (N:P:K) fertilizer and 50 t of composted manure were applied per ha. Drip irrigation was used with fertigation (1.4 dS m^−1^ EC value) at each irrigation using 1–2–1 fertilizer until first fruit set, 2–1-1 fertilizer until first fruit ripening and 1–1-2 fertilizer after first fruit ripening. Total genomic DNA was isolated from the leaf tissue bulked from 10 plants of the parental accessions and 93 individuals of the IBL population using a CTAB method [29]. Genomic DNA was quantified using Qubit™ quantitation assay (Life Technologies). DNA integrity was checked on a 1% agarose gel.

### Genotyping by sequencing (GBS)

Genomic DNA from the 93 individuals of the IBL population and parental accessions were subjected to GBS analysis by the University of Wisconsin Biotechnology Center (Madison, WI USA). Sequencing library preparation protocol, including restriction enzyme digestion, use of barcode adapters, sample pooling and amplification, was performed as described by Elshire et al. [18]. The 95-plex library was sequenced with the Illumina HiSeq next-generation sequencing platform (Illumina Inc. San Diego, CA).

### GBS data analysis and SNP identification

GBS data analysis was performed using the GBS discovery pipeline of TASSEL version 5.0 software [30]. The FASTQ and sample key files (containing the barcodes for each genotype) generated from raw sequence reads by the CASAVA 1.8.2 software package (Illumina Inc.) were used as input for processing in the pipeline. Before the analysis, 64-base reads were generated by trimming reads having the barcodes for each genotype followed by an *Ape*KI cut site using the FastqToTagCountPlugin of the pipeline. Reads with unidentified bases (N) were excluded from analysis. The barcoded sequence reads were collapsed into unique sequence tags with counts using the FastqToTagCountPlugin with default parameters with the exception that minimum number of times a tag must be present was set to 3. Tag count files that contained the sequence tags that passed the minimum count threshold of 3 were merged into a master file using the MergeMultipleTagCountPlugin. The master tags in FASTQ format generated by TagCountToFastqPlugin were aligned to the tomato *S. lycopersicum* reference genome using the bowtie2 plugin with default parameters [31, 32]. SAMConverterPlugin generated the “Tags On Physical Map” (TOPM) file which contained information about the physical positions of the master tags which had the best unique alignments with the reference genome. In addition to the TOPM file, the “Tags by Taxa” (TBT) file that contained tag counts of each barcode generated by FastqToTBTPlugin was used for SNP calling according to the parameters of the TagsToSNPByAlignmentPlugin (Additional file 1: Table S1). SNPs were recorded in a HapMap file for each chromosome. MergeDuplicateSNPsPlugin was used to merge the duplicate SNPs. SNPs were filtered based on minimum Taxon Coverage (mnTCov: 0.01), minimum Site Coverage (mnSCov: 0.2), linkage disequilibrium with neighboring SNPs (hLD: TRUE), minimum R2 value for the LD filter [−mnR2]: 0.2, and minimum Bonferroni-corrected *p*-value for the LD filter [−mnBonP]: 0.005. A physical map of the identified SNPs was drawn using Mapchart software [33].

### Phenotypic evaluation

Tomato fruits at the normal market stage were evaluated for 11 qualitative fruit quality traits: fruit weight, dry matter weight, external color, internal color, locule number, wall thickness, firmness, fruit shape, stem scar, total soluble solids content and pH. Phenotyping was performed as described in the first such work using *S. pimpinellifolium* [25] and most of the studies thereafter [1, 3, 6, 7]. Fruit weight (FW) was determined by bulking the fruit from 10 plants and calculating the mean weight of 10 representative tomato fruits. Fruits from 10 plants per genotype were bulked and characterized for external and internal color, fruit firmness, shape, stem scar, locule number, wall thickness and total soluble solids content. External (EXC) and internal fruit colour (INC) were visually determined for each individual using a scale from 1 to 5 (1 = yellow or orange, 5 = most intense red). A total of 100 g tomato fruits were dried and weighed to calculate fruit dry matter. Fruit firmness (FIRM) was determined by hand squeezing using a scale of 1 to 5 (1 = soft, 5 = very firm). Ratio of fruit length to fruit width represented fruit shape (FS) with a scale from 1 to 5 (1 = round, 5 = elongated). Stem scar diameter (1 = small, 5 = very large) represented stem scar size (SCAR). Locule number (LN) was counted in transversely-cut fruits. Fruit wall thickness (pericarp thickness) (WALL) was visually determined using a scale from 1 to 5 (1 = thin, 5 = very thick) [25]. Total soluble solids content of the tomato fruits was measured using a refractometer. The pH of juice from the fruits was measured with a pH meter.

### QTL mapping

QGene version 4.0 [34] was used for QTL analysis. The CIM (Composite Interval Mapping) QTL analysis method uses both interval mapping and multiple regression analysis and was performed with automatic forward cofactor selection and a scan interval of 0.2 Mb. A total of 1,000 random permutations were performed with parameters (a = 0.05) to calculate the genome-wide LOD threshold [35]. Correlation analysis between traits was performed using PASW software [36].

## Results

### GBS

A total of 120,983,088 reads were generated by sequencing of the 95-plex library. From these reads, 448,539 sequence tags were generated. A majority of the sequence tags (84.4%, 378,659) were uniquely aligned to the tomato genome. The remaining tags were either aligned to multiple positions (13.8%, 61,793) or could not be located (1.8%, 8,087) to the tomato genome assembly. The 378,659 sequence tags uniquely aligned to the genome were used for genome-wide high-throughput SNP discovery. Sequences of the merged GBS tags in SAM (Sequence Alignment/Map) file format can be accessed at SRA (Sequence Read Archive) database under accession number SRP078914.

### SNP identification

**Fig. 1:**
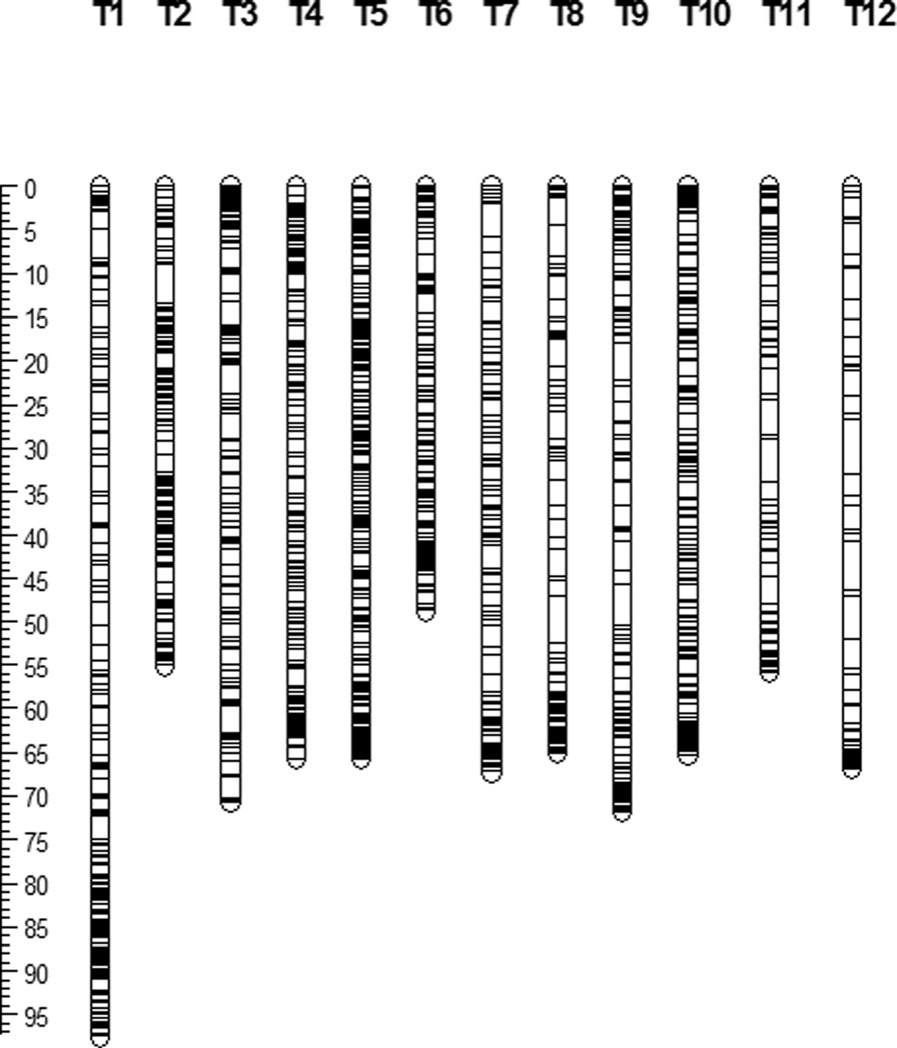
Physical map of the 3,125 SNPs retained after the filtering process. Detailed information about the physical map and SNP locations is available in the Additional file 2

Tag alignment to the reference genome revealed 23,677 unique SNP loci (pre-filtration SNPs) between the *S. lycopersicum* and *S. pimpinellifolium* genomes. The SNP loci were found on all 12 chromosomes of tomato (T1-T12). The physical map constructed with the identified SNP loci had sufficient coverage for genome analysis such as QTL mapping and contained SNPs that were evenly distributed along the chromosomes with 63 gaps in size of more than 2 Mb (Fig. 1). After filtration based on the proportion of missing data (less than 20%) and parameters such as minimum Taxon Coverage (mnTCov: 0.01), minimum Site Coverage (mnSCov: 0.2), linkage disequilibrium with neighboring SNPs (hLD: TRUE), minimum R^2^ value for the LD filter [−mnR2]: 0.2, minimum Bonferroni-corrected p-value for the LD filter [−mnBonP]: 0.005, a total of 3,125 SNP loci were retained. While the average distance between adjacent loci was 33.8 kb for the pre-filtration SNPs, frequency was reduced to one SNP per 256.4 kb after filtering (Table 1). Chromosome T6 had the highest frequency of filtered SNPs with an average distance of 129.7 kb between adjacent markers (Table 1).

**Table 1:** Numbers and frequencies of pre-filtration and filtered SNP loci in tomato *S. lycopersicum* and *S. pimpinellifolium* genomes

		Number of SNPs		Frequency (kb) of SNPs	
Chromosome	Size of LG (Mb)	Pre-filtration^a^	Filtered^b^	Pre-filtration^a^	Filtered^b^
T1	98.4	2193	293	44.9	335.9
T2	55.2	1773	297	31.2	186.0
T3	70.8	1979	200	35.8	353.8
T4	66.4	1816	271	36.6	245.1
T5	65.9	2002	442	32.9	149.0
T6	49.5	3521	382	14.1	129.7
T7	68.0	1551	213	43.9	319.4
T8	65.8	1360	176	48.4	373.9
T9	72.4	2664	299	27.2	242.2
T10	65.5	1503	299	43.6	219.1
T11	56.2	2430	165	23.1	340.8
T12	67.1	885	88	75.8	762.1
Total	801.3	23677	3125	33.8	256.4

^a^Number and frequency of SNPs physically mapped in tomato genome

^b^Number and frequency of SNPs retained after the filtration process

The majority of the SNPs (56.2%) identified in this study were transition mutations (A/G or C/T) as expected. The most frequently observed substitution types, A/G and C/T transitions, had similar frequencies: 28.2 and 28.0%, respectively. C/G transversion was the least common substitution type (7.9%) (Additional file 1: Table S2). The observed transition/transversion ratio was 1.28.

### Phenotypic variation

A total of 93 IBL individuals and parental accessions were characterized for 11 fruit quality traits including fruit weight, dry matter weight, external and internal color, locule number, wall thickness, firmness, fruit shape, stem scar, soluble solids content and pH. The parents of the IBL population had extreme phenotypes for fruit weight, wall thickness, stem scar and soluble solids content traits (Additional file 1: Figure S1 and Table 2). All of the traits segregated in the IBL population. Fruit weight, wall thickness, firmness and stem scar traits displayed the highest variation in the population with coefficients of variation (CV) ranging from 33.5 to 46.4%, (Table 2). With the exception of soluble solids content and pH, the remaining traits (dry weight, external color, internal color, locule number and fruit shape) had considerable variation in the population (CVs ranging from 21.2 to 27.5%). The pH and soluble solids contents displayed the lowest variation with 7 and 9.4% CV, respectively (Table 2). All traits except external color, locule number and fruit shape displayed normal and continuous distributions (Additional file 1: Figure S1).

**Table 2:** Statistics for fruit quality traits measured in IBL population and parents; *S. lycopersicum* cv. Tueza and *S. pimpinellifolium* cv. LA1589

	Parents		IBL population		
Fruit traits	Tueza	LA1589	Mean	Range	CV%
Fruit weight (g)	118.4	0.8	65.5 ± 3.1	10.4–190.2	46.4
Dry matter weight (g)	4.6	5.2	5.1 ± 0.1	1.3–8.4	21.2
External color (1–5)	3	5	4.0 ± 0.1	0–5.0	21.6
Internal color (1–5)	4	3	3.3 ± 0.1	0–5.0	27.5
Locule number (1–5)	3	2	3.2 ± 0.1	0–4.0	24.6
Wall thickness (1–5)	3.5	1	2.8 ± 0.1	0–5.0	42.6
Firmness (1–5)	3	3.5	3.1 ± 0.1	0–5.0	33.9
Fruit shape (1–5)	1	1	1.0 ± 0	0–2.0	22.5
Stem scar (1–5)	4	1	3.1 ± 0.1	0–5.0	33.5
Soluble solids content	4.4	8.2	5.2 ± 0.05	4–6.8	9.4
pH	4	4	4.0 ± 0	3.7–6	7

Correlation analysis demonstrated significant associations between some traits. Fruit weight was correlated to all traits except dry matter weight, internal color, fruit shape and pH. Fruit weight was positively correlated to locule number (r^2^ = 0.40) and wall thickness (r^2^ = 0.50) and negatively weakly correlated to external color (r^2^ = −0.27) and soluble solids content (r^2^ = −0.26). Dry matter weight was moderately correlated to soluble solids content (r^2^ = 0.47). External color was weakly correlated to internal color (r^2^ = 0.38) and locule number (r^2^ = 0.25). Locule number was moderately correlated to stem scar (r^2^ = 0.55) (Additional file 1: Table S3).

### QTL mapping

The 3,125 genome-wide SNP loci (Fig. 1) that were retained after filtering were used in QTL mapping of the fruit quality traits. CIM analysis was performed and a logarithm of odds (LOD) threshold (*p* < 0.05) generated by 1,000 permutations was used to identify QTLs for each trait. For fruit weight, the LOD threshold was 3.1 (Additional file 1: Table S4) and three QTLs (*fw2.1*, *fw4.1* and *fw6.1*) were identified on chromosomes T2, T4 and T6 (Table 3). The percentage of phenotypic variation (PVE) explained by the QTLs varied from 15 to 26% (Table 3). The QTL on chromosome T4 (*fw4.1*) had the highest PVE, 26%. Three QTLs were identified for dry matter weight on chromosome T7 based on a LOD threshold of 3.3. The PVEs of these loci were 19, 15 and 14% for *dw7.1*, *dw7.2* and *dw7.3*, respectively (Table 3).

**Table 3:** QTLs identified for 11 fruit quality traits

Trait	QTL	Chr.	Position (Mb)^a^	Marker interval	LOD	PVE^b^	Additive
							effect^c^
Fruit weight	fw2.1	T2	51.6–52	SpimpSNP_chr2_51653038 - SpimpSNP_chr2_52236461	3.1	15	*S. pimpinellifolium*
Fruit weight	fw4.1	T4	22.5–22.9	SpimpSNP_chr4_21588199 - SpimpSNP_chr4_23188806	5.6	26	*S. lycopersicum*
Fruit weight	fw6.1	T6	24.2–19.8	SpimpSNP_chr6_23671779 - SpimpSNP_chr6_24889074	3.6	17	*S. lycopersicum*
Dry matter weight	dw7.1	T7	6–7.8	SpimpSNP_chr7_2225863 - SpimpSNP_chr7_9627011	4.1	19	*S. lycopersicum*
Dry matter weight	dw7.2	T7	27.8–29.4	SpimpSNP_chr7_26481282 - SpimpSNP_chr7_28434174	3.2	15	*S. lycopersicum*
Dry matter weight	dw7.3	T7	39.6–44.8	SpimpSNP_chr7_39834929 - SpimpSNP_chr7_44678356	3	14	*S. lycopersicum*
External color	exc1.1	T1	67.6–67.8	SpimpSNP_chr3_67613866 - SpimpSNP_chr3_67813317	3	11	*S. pimpinellifolium*
External color	exc2.1	T2	62.3–62.5	SpimpSNP_chr4_62352850 - SpimpSNP_chr4_62544061	3	10	*S. pimpinellifolium*
Internal color	inc2.1	T2	23.6–23.8	SpimpSNP_chr2_23655570 - SpimpSNP_chr2_24268112	4.9	23	*S. pimpinellifolium*
Internal color	inc2.2	T2	34.8	SpimpSNP_chr2_34886535–SpimpSNP_chr2_35242658	4.5	20	*S. pimpinellifolium*
Internal color	inc2.3	T2	2–3.8	SpimpSNP_chr2_640497–SpimpSNP_chr2_3894978	5	20	*S. pimpinellifolium*
Internal color	inc4.1	T4	16.5–16.7	SpimpSNP_chr4_16565256 - SpimpSNP_chr4_18177074	3.4	15	*S. pimpinellifolium*
Internal color	inc6.1	T6	30.4	SpimpSNP_chr6_30399172 - SpimpSNP_chr6_31009885	3.1	14	*S. lycopersicum*
Internal color	inc7.1	T7	34.3–34.4	SpimpSNP_chr7_33799287 - SpimpSNP_chr7_34463608	4.2	16	*S. lycopersicum*
Internal color	inc8.1	T8	10.4–11	SpimpSNP_chr8_10421348 - SpimpSNP_chr8_15081462	3.8	17	*S. pimpinellifolium*
Internal color	inc10.1	T10	51.4	SpimpSNP_chr10_51446730 - SpimpSNP_chr10_53466408	5.5	24	*S. pimpinellifolium*
Internal color	inc12.1	T12	23.7–24.1	SpimpSNP_chr12_21186959 - SpimpSNP_chr12_24152718	5.2	23	*S. lycopersicum*
Locule number	ln2.1	T2	47.2–51.4	SpimpSNP_chr2_47074933 -SpimpSNP_chr2_51653038	7	30	*S. lycopersicum*
Locule number	ln4.1	T4	5.7	SpimpSNP_chr4_5137285 - SpimpSNP_chr4_6526895	3.8	13	*S. lycopersicum*
Wall thickness	wall10.1	T10	21.6–22.6	SpimpSNP_chr10_19888032 - SpimpSNP_chr10_23051275	3.4	15	*S. lycopersicum*
Wall thickness	wall12.1	T12	62.5	SpimpSNP_chr12_52326486 - SpimpSNP_chr12_63747215	3	13	*S. lycopersicum*
Firmness	firm1.1	T1	2.8–3	SpimpSNP_chr1_2881522 - SpimpSNP_chr1_8892676	3.5	14	*S. pimpinellifolium*
Firmness	firm1.2	T1	80.2–80.4	SpimpSNP_chr1_79834277 - SpimpSNP_chr1_80687203	3.8	18	*S. pimpinellifolium*
Firmness	firm4.1	T4	2.5	SpimpSNP_chr4_1705692 - SpimpSNP_chr4_2593932	4	19	*S. pimpinellifolium*
Firmness	firm10.1	T10		SpimpSNP_chr10_1689980 - SpimpSNP_chr10_2014825	5.3	24	*S. pimpinellifolium*
Fruit shape	fs4.1	T4	5.7	SpimpSNP_chr4_5717067 - SpimpSNP_chr4_6526895	6,1	26	*S. lycopersicum*
Fruit shape	fs10.1	T10	11.2–11.8	SpimpSNP_chr10_10418801 -SpimpSNP_chr10_12085921	5.2	14	*S. lycopersicum*
Fruit shape	fs12.1	T12	33.1–35.5	SpimpSNP_chr12_33126847 - SpimpSNP_chr12_36718114	5.5	20	*S. lycopersicum*
Fruit shape	fs12.2	T12	59.7–62.7	SpimpSNP_chr12_52326486 - SpimpSNP_chr12_63747215	5.2	23	*S. lycopersicum*
Stem scar	sc7.1	T7	64.4	SpimpSNP_chr7_64272106 - SpimpSNP_chr7_64876647	3.6	16	*S. lycopersicum*
Stem scar	sc10.1	T10	29.8–35.6	SpimpSNP_chr10_36881278 - SpimpSNP_chr10_29632232	3.3	3	*S. pimpinellifolium*
Soluble solids content	ssc1.1	T1	26.6–27.2	SpimpSNP_chr1_24085783 - SpimpSNP_chr1_27278048	5.3	23	*S. pimpinellifolium*
Soluble solids content	ssc2.1	T2	36.6–38.6	SpimpSNP_chr2_36642750 - SpimpSNP_chr2_38643210	8.2	34	*S. pimpinellifolium*
Soluble solids content	ssc8.1	T8	60.2–61.2	SpimpSNP_chr8_60424301 - SpimpSNP_chr8_61250753	3.7	17	*S. lycopersicum*
Soluble solids content	ssc10.1	T10	22.8	SpimpSNP_chr10_23051275 - SpimpSNP_chr10_19888032	3.6	17	*S. lycopersicum*
pH	ph1.1	T1	66.8	SpimpSNP_chr1_65817896 - SpimpSNP_chr1_67006382	12	47	*S. lycopersicum*
pH	ph8.1	T8	63.8	SpimpSNP_chr8_63683606 - SpimpSNP_chr9_755740	6.4	14	*S. lycopersicum*

^a^Peak position of QTL

^b^Percentage of phenotypic variation explained by identified QTL

^c^Parental allele associated with increased trait value

A total of 11 QTLs were identified for external and internal color in tomato based on LOD thresholds of 3.1 and 3, respectively (Additional file 1: Table S4). For the external color, two QTLs were identified on chromosomes T1 and T2 with a total PVE of 21%. A total of nine QTLs were identified for internal color on chromosomes T2, T4, T6, T7, T8, T10, and T12. The percentage of phenotypic variation (PVE) explained by the loci varied from 14 to 24% (Table 3).

Two QTLs were identified for locule number on chromosomes T2 and T4 with a LOD threshold of 3.6 (Additional file 1: Table S4). PVEs of the QTLs were 30% for *ln2.1* and 13% for *ln4.1* (Table 3). LOD threshold for wall thickness was 3 (Additional file 1: Table S4) and two QTLs were identified on chromosomes T10 and T12. PVEs of the QTLs were 15 and 13%, for *wall10.1* and *wall12.1*, respectively.

A total of four QTLs with LOD scores higher than the threshold (3.2) were identified for firmness on chromosomes T1, T4 and T10. PVEs of the QTLs varied from 14 to 24% (Additional file 1: Table S4 and Table 3). For fruit shape, four QTLs were detected on chromosomes T4, T10 and T12 (with LOD scores greater than the threshold 5) (Additional file 1: Table S4 and Table 3). PVEs of the QTLs for the trait ranged from 14 to 26%. The QTL on chromosome T4 had the highest PVE (26%) (Table 3). Two QTLs were determined for stem scar (LOD greater than 3) on chromosomes T7 and T10 (Additional file 1: Table S4, Table 3). PVEs of the QTLs were 16 and 3% for *sc7.1* and *sc10.1*, respectively (Table 3).

LOD thresholds for soluble solids content and pH traits were 3.1 and 6.2, respectively (Additional file 1: Table S4). For soluble solids content, four QTLs were identified on chromosomes T1, T2, T8 and T10. PVEs of the QTLs varied from 17 to 34%. The QTL on chromosome T2 had the highest PVE (34%) (Table 3). For pH, two QTLs were identified on chromosomes T1 and T8. The QTL on chromosome T1 had a major allelic effect with a PVE of 47%. The PVE of the QTL on chromosome T8 was 14% (Table 3).

Colocalization of the QTLs indicates that a given QTL has an effect on more than one trait. In the present study, a few colocalized QTLs were detected. QTLs on chromosomes T2 and T4 for locule number colocalized with QTLs for fruit weight and fruit shape, respectively. QTLs on chromosomes T10 and T12 for wall thickness colocalized with QTLs for soluble solids content and fruit shape, respectively. QTLs for pH and external color colocalized on chromosome T1 (Additional file 1: Table S5).

QTL mapping revealed several *S. pimpinellifolium* alleles potentially useful for improving fruit traits. A total of 16 QTLs for traits such as fruit weight, external and internal color, firmness, soluble solids content and stem scar had favourable alleles provided by the *S. pimpinellifolium* parent. *S. pimpinellifolium* had the highest breeding potential for internal color with six QTLs on chromosomes T2, T4, T8 and T10 having *S. pimpinellifolium* alleles that improved the trait (PVEs of the QTLs ranged between 15 and 24%). All the QTLs for external color and firmness on chromosomes T1, T2, T4 and T10 had favourable *S. pimpinellifolium* alleles with total PVEs of 21 and 75%, respectively. For soluble solids content, S. *pimpinellifolium* alleles improved the trait for two QTLs, with PVEs of 23% and 34% (*ssc1.1* and *ssc2.1*). Also single QTLs on chromosomes T2 and T10 for fruit weight and stem scar, respectively, had wild alleles which improved the traits = with PVEs of 15% and 3%, (*fw2.1, sc10.1*).

## Discussion

### SNP identification by GBS analysis

The analysis of GBS data in conjunction with a well-established reference genome is a relatively straightforward route for SNP calling and marker ordering along chromosomes [37]. In this work, most of the sequence tags (84.4%) could be uniquely aligned to the tomato reference genome. This was expected because complete genome assemblies of tomato are available.

In the present study, although 23,677 SNPs were discovered by comparison of the *S. lycopersicum* and *S. pimpinellifolium* genome sequences, most of the SNPs (80%) were excluded due to a high proportion of missing data. Nevertheless, a sufficient number of validated SNP markers (3,125 SNPs) remained and were found to be useful for QTL mapping. In comparison with previous work, this study discovered fewer validated SNPs than are available in The Solanaceae Genomics Network database (9,226 SNPs) and those reported by Chen et al. [15] (4,697 SNPs). The current number of SNPs is also much lower than discovered by Causse et al. [16] (16,000 SNPs) and Kim et al. [17] (4,680,647); however, these SNPs were not validated. The average frequency of SNPs identified in this study was 1 SNP per 256.4 kb, much higher than reported for the *S. lycopersicum* × *S. pimpinellifolium* linkage maps of Salinan et al. [13] (1 SNP per 8,482 kb), Capel et al. [14] (1 SNP per 4,077 kb) and Chen et al. [15] (1 SNP per 1,821 kb). Thus, the present research demonstrated that the GBS approach was efficient in constructing a SNP-based physical map of sufficient resolution for QTL mapping in tomato.

### Phenotypic variation

The IBL population and parental genotypes were evaluated for 11 fruit quality traits in order to identify associated QTLs. Sizable variation for all traits except soluble solids content and pH, and normal continuous distribution of all but three traits (external color, locule number and fruit shape) were observed in the IBL population. External color and locule number tended to skew toward more intense red color and higher locule numbers due to the unbalanced nature of the IBL population which favors the recurrent parent genotype. The parents of the IBL population had extreme alleles for fruit weight, wall thickness, stem scar and soluble solids content traits (Table 2). Although the parental alleles for soluble solids content were extreme, low variation was observed in the IBL population for the trait. This finding implies an unbalanced introgression of *S. pimpinellifolium* alleles for soluble solids content into the *S. lycopersicum* genome.

The present study demonstrated correlations between fruit quality traits, however, most of the significant correlations were weak. Correlations between fruit weight and all traits except fruit shape, internal color, dry matter weight and pH demonstrated that fruit weight was associated with fruit quality traits such as locule number, wall thickness, firmness and stem scar. Fruit weight had a high positive correlation with locule number. This is expected because increased locule number has a direct effect on fruit size and weight. Negative correlations of fruit weight with external color and soluble solids content indicate that intensity of external color decreases with increased fruit size due to decreased lycopene content and that sucrose content is negatively correlated with fruit volume. This negative correlation was also reported by Chen et al. [1], Doganlar et al. [3], Sun et al. [5] and Fulton et al. [39]. Correlation results between fruit weight and quality traits were consistent with the results of Lippman and Tanksley [2], Okmen et al. [38] and Fulton et al. [39]. A direct effect of soluble solids content on dry matter weight was observed in the IBL population. The positive correlation between internal color and external color was expected and consistent with previous reports [38–40]. These correlations can also be attributed to the pleiotropic effects of genes on different fruit quality traits.

### QTL mapping

Fruit quality parameters are important agronomic traits that increase the market value of both fresh market and processing tomatoes. Thus, there are many reports on QTL identification for fruit quality traits. All previous QTL mapping studies were performed using low density linkage maps constructed with PCR-based markers (SSR and COSII) and RFLP probes. Various parental lines and mapping populations such as BC_2_F_2_, IBL and RIL were used in these previous studies. This is the first study in which QTLs for fruit quality traits were identified by constructing a high density SNP-based physical map using a recently developed IBL population that carries introgressions from the *S. pimpinellifolium* genome. The physical map of SNP markers was useful for QTL mapping as IBLs are unbalanced populations which are not suitable for linkage map construction.

Fruit weight is the focus of many studies because increased fruit weight has direct effects on tomato yield [1–3, 14, 26, 27]. Fruit size is also an important trait that directs consumer preferences. Medium and large tomatoes are usually preferred by consumers [41]. In this work, three QTLs were identified on chromosomes T2, T4 and T6 for fruit weight. Previous studies identified three major and two minor QTLs on chromosomes T1, T2, T3, T7 and T11. Although QTL locations varied among these studies, all studies identified a QTL with major effect on chromosome T2 corresponding to a cloned gene that controls fruit weight (*fw2.2*) [42]. In the present study, the fruit weight QTL on chromosome T2 explained a variance of 15% for the trait, a value which is relatively low when compared with the same QTL in other studies (PVEs ranged between 15 and 40%). Differences in QTL magnitudes of effect and locations are most likely due to differences in population type used in the studies. The present work is most similar to the work of Doganlar et al. [3] which also studied an IBL population, which used a processing tomato as the recurrent parent. PVE of the QTL on chromosome T2 was the same as that reported by Doganlar et al. [3] (15%) due to the similarity of the genetic structures of the populations (IBL) used in the two studies. Identification of previously undetected QTLs on chromosomes T4 and T6 in the present work can be attributed to variation in the genetic backgrounds of the two mapping populations which is due to the use of different recurrent parents.

Because dried tomatoes have a high economic value, fruit dry matter weight can be as important as fruit weight. A previous QTL mapping study performed by Saliba-Colombani et al. [26] identified QTLs (with PVEs ranging from 9 to 25%) on chromosomes T2, T4 and T9 in a RIL population developed from the cross between a cherry tomato cultivar and *S. lycopersicum*. In other work, QTLs were identified on chromosomes T8, T10, T11 and T12 using 20 introgression lines carrying *S. chmielewski* introgressions in a *S. lycopersicum* genetic background [27]. In the present study, none of the above mentioned QTLs were detected. This result can be due to insufficient variation for dry matter weight between the parents and the moderate coefficient of variation detected for the trait in the mapping population. PVEs of identified QTLs ranged from 14 to 19%, suggesting that in contrast to fruit weight, dry matter weight is not controlled by major effect QTLs.

In the present study, while a total of nine QTLs were identified for internal color, only two loci were identified for external color. The low number of QTLs identified for external color might be due to the unbalanced segregation of the trait in the IBL population. Previous work detected QTLs for external color on chromosomes T1, T3, T4, T7, T8, T9, T11 and T12 [38.43]. Although a QTL was also identified on chromosome T1 in this work, the physical position of the closest marker (C2_At5g13030: 1.1 Mb) to the locus on the same chromosome by Okmen et al. [38] reveals that the two QTLs are not identical. For internal color, previous studies identified QTLs on chromosomes T1, T3, T4, T7, T8, T9 and T12 with PVEs that ranged between 5 to 30% [7, 38]. In the present work, QTLs for internal color were identified on chromosomes T4, T7 and T8. The physical positions of the markers (65.4 Mb, 55 Mb and 58.1 Mb for At1g47830, T0671 and TG307, respectively) linked to the three QTLs indicated that they do not overlap with the QTLs identified in previous work.

Previous studies showed that locule number is controlled by six QTLs on chromosomes T2, T3, T4, T7, T10 and T12 [38, 43]. In addition, a major gene for locule number was mapped at the 48.1 Mb position on chromosome T2 [44]. The major QTL (*ln2.1*) containing this single gene (*lc*) was also identified in the present study (PVE of 30%). In addition to this major QTL, a new QTL with minor effect was identified on chromosome T4.

Wall thickness and firmness are important fruit quality traits that define the shelf life of tomatoes. QTLs with minor effects on wall thickness were reported on chromosomes T6, T8, T11 and T12 [38], however, these loci do not overlap with those reported in the present work. Previously, QTLs for firmness were identified on chromosomes T1, T2, T3, T4, T5, T8 and T10 [3, 38]. In addition to these previously identified QTLs, four new QTLs were identified for firmness trait in this work.

Fruit shape and stem scar are appearance traits analysed in this study. Globular fruits with small stem scar are favoured in the market. More than 10 QTLs for fruit shape were identified in previous studies [3, 28, 38]. In addition to these QTLs, a total of four new QTLs were identified in this work with minor effects on fruit shape. For stem scar, seven QTLs were previously identified in tomato [2, 3, 38]. One of the two QTLs identified in this study for the stem scar was previously reported at 65.5 Mb position on chromosome T7 with a low PVE of 8% [3].

Soluble solids content and pH are important traits for fresh market tomatoes as they help define flavor [45]. A total of five QTLs were detected on chromosomes T1, T6, T8 and T9 in previous studies for soluble solids content [1, 3, 14, 26] The present report demonstrated that different QTLs (chromosome T1, T2, T8 and T10) control soluble solids content in fresh market tomatoes. For pH, a total of six QTLs were identified in tomato on chromosomes T1, T2, T4, T5, T9 and T12 in previous studies [1, 14, 26]. While the position of the previously identified QTL on chromosome T1 [1] was at 86 Mb, the major effect QTL (47%) identified on the same chromosome in this work was positioned at 66.8 Mb. Thus, the QTL identified in this study is close to the QTL previously identified by Chen et al. [1]. These two QTLs might actually overlap because the SNP-based map of the present study has much higher resolution than the linkage map of Chen et al. [1].

Some QTLs colocalized as expected. For example, QTLs for locule number coincided with those for fruit weight and fruit shape because increased locule number results in larger tomatoes. However, colocalization of a QTL for pH with one for external color and colocalization of loci for wall thickness and soluble solids content were unexpected. These unexpected colocalizations might be due to linkage of the genes that control the traits [46].

This present study confirmed the high breeding potential of *S. pimpinellifolium* by detecting useful alleles for breeding of fruit quality traits such as fruit weight, external and internal color, firmness, soluble solids content and stem scar. The findings were expected for external color and soluble solids content because *S. pimpinellifolium* had higher values than cultivated tomato. In contrast, although *S. pimpinellifolium* had lower values than *S. lycopersicum* for fruit weight, internal color and stem scar*,* favorable *S. pimpinellifolium* alleles were detected for these traits. This result was consistent with the work of Top et al. [6]. In that study, although *S. pimpinellifolium* had lower values than cultivated tomato for fruit weight and firmness, some individuals from an IBL (BC_2_F_9_) population derived from the cross *S. lycopersicum* and *S. pimpinellifolium* (LA1589) showed higher values than *S. lycopersicum* (TA209)*.*

## Conclusions

The present research demonstrated that GBS was an efficient technique for construction of a SNP-based physical map with sufficient resolution for mapping fruit quality QTLs in tomato. The identified SNPs were well distributed in the tomato genome. This study also revealed valuable *S. pimpinellifolium* alleles for most of the traits. Thus, in this work, valuable findings were obtained for unlocking the genetic potential of this wild species for tomato breeding.

## Additional files

Additional file 1: Table S1. TASSEL software parameters used for SNP calling and filtering. **Table S2.** Types of substitutions represented by the identified SNP loci. **Table S3.** Significant (*P* < 0.05) correlations between tomato fruit traits. Correlations with *P* value > 0.05 were considered to be non-significant (NS). FW = Fruit weight, DW = Dry matter weight, EXC = External color, INC = Internal color, LN = Locule number, WALL = Wall thickness, FIRM = Firmness, FS = Fruit shape, SCAR = Stem scar, SSC = Soluble solids content. **Table S4.** LOD thresholds of 11 fruit quality traits calculated by 1,000 random permutations with parameters (a = 0.05). **Table S5.** Fruit quality QTLs that colocalized. **Figure S1.** Frequency distributions for the fruit traits in the IBL population. FW = Fruit weight, DW = Dry matter weight, EXC = External color, INC = Internal color, LN = Locule number, WALL = Wall thickness, FIRM = Firmness, FS = Fruit shape, SCAR = Stem scar, SSC = Soluble solids content. All the traits except external color, locule number and fruit shape showed normal and continuous distribution (DOCX 74 kb)
Additional file 2: Additional excel file containing physical maps of identified and filtered SNPs and the linkage map. (XLSX 165 kb)

## Abbreviations

AB: Advanced backcross
CIM: Composite interval mapping
COSII: Conserved ortholog Set II
CV: Coefficients of variation
DW: Dry matter weight
EXC: External color
FIRM: Firmness
FS: Fruit shape
FW: Fruit weigh
GBS: Genotyping by sequencing
HRM: High-resolution melting
IBL: Inbred backcross lines
INC: Internal color
LN: Locule number
NGS: Next-generation sequencing
PVE: Percentage of phenotypic variation explained by the QTL
QTL: Quantitative trait loci
RFLP: Restriction fragment length polymorphism
SCAR: Stem scar
SNP: Single nucleotide polymorphism
SSC: Soluble solids content
SSR: Simple sequence sepeats
TBT: Tags by taxa
TOPM: Tags on physical map
WALL: Wall thickness
