# Supplementary material for: Genome-wide SNP discovery and QTL mapping for fruit quality traits in inbred backcross lines (IBLs) of solanum pimpinellifolium using genotyping by sequencing
Source: BMC Genomics. 2017 Jan 3;18:1. doi: 10.1186/s12864-016-3406-7 (PMC5209891; doi:10.1186/s12864-016-3406-7)
Supplement: Additional file 1: Table S1. — TASSEL software parameters used for SNP calling and filtering. Table S2. Types of substitutions represented by the identified SNP loci. Table S3. Significant (P < 0.05) correlations between tomato fruit traits. Correlations with P value > 0.05 were considered to be non-significant (NS). FW = Fruit weight, DW = Dry matter weight, EXC = External color, INC = Internal color, LN = Locule number, WALL = Wall thickness, FIRM = Firmness, FS = Fruit shape, SCAR = Stem scar, SSC = Soluble solids content. Table S4. LOD thresholds of 11 fruit quality traits calculated by 1,000 random permutations with parameters (a = 0.05). Table S5. Fruit quality QTLs that colocalized. Figure S1. Frequency distributions for the fruit traits in the IBL population. FW = Fruit weight, DW = Dry matter weight, EXC = External color, INC = Internal color, LN = Locule number, WALL = Wall thickness, FIRM = Firmness, FS = Fruit shape, SCAR = Stem scar, SSC = Soluble solids content. All the traits except external color, locule number and fruit shape showed normal and continuous distribution (DOCX 74 kb) [file 12864_2016_3406_MOESM1_ESM.docx]

Table S1. TASSEL software parameters used for SNP calling and filtering.

| Parameter | Value | Comment |
| --- | --- | --- |
| mnMAC | 100000 | Minimum minor allele count ( pass = mnMAF or mnMAC ) for SNP calling |
| misMat | 0.1 | Threshold genotypic mismatch rate above which the duplicate SNPs will not be merged. |
| mnTCov | 0.01 | Minimum taxon coverage. The minimum SNP call rate for a taxon to be included in the output |
| mnSCov | 0.2 | Minimum site coverage. The minimum taxon call rate for a SNP to be included in the output |
| mnMAF | 0.01 | Minimum minor allele frequency |
| mnR2 | 0.2 | Minimum R-square value for the LD filter. |
| mnBonP | 0.005 | Minimum Bonferroni-corrected p-value for the LD filter. |
| hLD | TRUE | whether or not filtered for those in statistically significant LD with at least one neighboring SNP |

Table S2. Types of substitutions represented by the identified SNP loci.

| Transition/ Transversion | Number of SNPs | Frequency (%) |
| --- | --- | --- |
| Transition |  |  |
| C/T | 6619 | 28.0 |
| A/G | 6679 | 28.2 |
| Total | 13298 | 56.2 |
| Transversion |  |  |
| C/G | 1862 | 7.9 |
| A/C | 2892 | 12.2 |
| G/T | 2858 | 12.1 |
| A/T | 2767 | 11.7 |
| Total | 10379 | 43.8 |

Table S3. Significant (*P* < 0.05) correlations between tomato fruit traits. Correlations with *P* value > 0.05 were considered to be non-significant (NS). FW = Fruit weight, DW = Dry matter weight, EXC = External color, INC = Internal color, LN = Locule number, WALL = Wall thickness, FIRM = Firmness, FS = Fruit shape, SCAR = Stem scar, SSC = Soluble solids content.

| Traits |  | FW | DW | EXC | INC | LN | WALL | FIRM | FS | SCAR | SSC | pH |
| --- | --- | --- | --- | --- | --- | --- | --- | --- | --- | --- | --- | --- |
|  |  |  |  |  |  |  |  |  |  |  |  |  |
| FW |  | 1 | NS | -.27 | NS | .40 | .50 | .31 | NS | .32 | -.26 | NS |
| DW |  |  | 1 | .33 | .30 | NS | NS | NS | NS | NS | .467 | NS |
| EXC |  |  |  | 1 | .38 | .25 | NS | NS | NS | NS | NS | NS |
| INC |  |  |  |  | 1 | .26 | .24 | NS | NS | .37 | NS | NS |
| LN |  |  |  |  |  | 1 | .25 | NS | NS | .55 | NS | NS |
| WALL |  |  |  |  |  |  | 1 | .20 | .30 | .33 | NS | NS |
| FIRM |  |  |  |  |  |  |  | 1 | .15 | NS | NS | NS |
| FS |  |  |  |  |  |  |  |  | 1 | NS | NS | NS |
| SCAR |  |  |  |  |  |  |  |  |  | 1 | NS | NS |
| SSC |  |  |  |  |  |  |  |  |  |  | 1 | NS |
| pH |  |  |  |  |  |  |  |  |  |  |  | 1 |
|  |  |  |  |  |  |  |  |  |  |  |  |  |


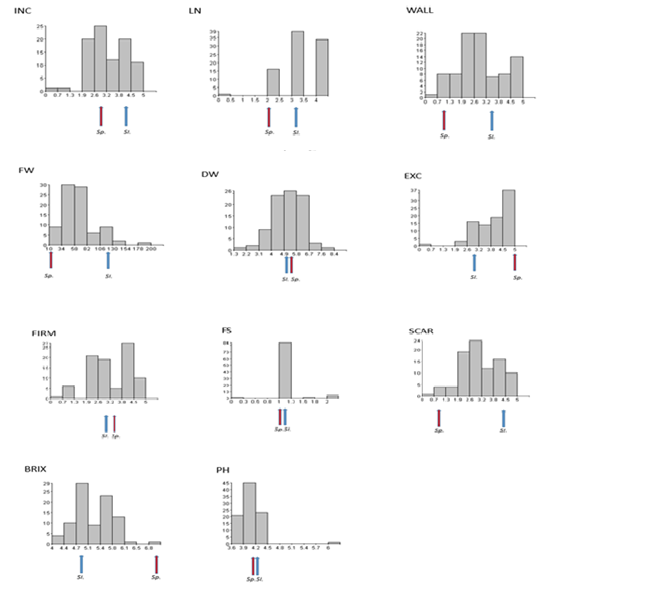


Figure S1. Frequency distributions for the fruit traits in the IBL population. FW = Fruit weight, DW = Dry matter weight, EXC = External color, INC = Internal color, LN = Locule number, WALL = Wall thickness, FIRM = Firmness, FS = Fruit shape, SCAR = Stem scar, SSC = Soluble solids content. All the traits except external color, locule number and fruit shape showed normal and continuous distribution.

Table S4. LOD thresholds of 11 fruit quality traits calculated by 1,000 random permutations with parameters (a = 0.05).

| Trait | LOD threshold |
| --- | --- |
| Fruit weight | 3.1 |
| Dry matter weight | 3.3 |
| External color | 3.1 |
| Internal color | 3 |
| Locule number | 3.6 |
| Wall thickness | 3 |
| Firmness | 3.2 |
| Fruit shape | 5 |
| Stem scar | 3 |
| Soluble solids content | 3.1 |
| pH | 6.2 |

Table S5. Fruit quality QTLs that colocalized.

| Trait | QTL | Chr. | Position* |
| --- | --- | --- | --- |
|  |  |  |  |
| pH | ph1.1 | T1 | 66.8 |
| External color | exc1.1 | T1 | 67.6 - 67.8 |
| Locule number | ln2.1 | T2 | 47.2 -51.4 |
| Fruit weight | fw2.1 | T2 | 51.6 - 52 |
| Locule number | ln4.1 | T4 | 5.7 |
| Fruit shape | fs4.1 | T4 | 5.7 |
| Soluble solids content | ssc10.1 | T10 | 22.8 |
| Wall thickness | wall10.1 | T10 | 21.6 - 22.6 |
| Wall thickness | wall12.1 | T12 | 62.5 |
| Fruit shape | fs12.2 | T12 | 59.7 -62.7 |
